# Fumarprotocetraric acid and geraniin were identified as novel inhibitors of human respiratory syncytial virus infection *in vitro*

**DOI:** 10.3389/fcimb.2024.1484245

**Published:** 2024-12-24

**Authors:** Chao Wang, Yi-Man Huang, Jun Zhao, Yi-Ming Bai, Cai-Qin Yan, Guan-Hua Du, Li-Shu Zheng, Ai-Lin Liu

**Affiliations:** ^1^ State Key Laboratory of Bioactive Substances and Functions of Natural Medicines, Institute of Materia Medica, Chinese Academy of Medical Sciences and Peking Union Medical College, Beijing, China; ^2^ Beijing Key Lab of Drug Target Identification and Drug Screening, Institute of Materia Medica, Chinese Academy of Medical Sciences & Peking Union Medical College, Beijing, China; ^3^ National Key Laboratory of Intelligent Tracking and Forecasting for Infectious Diseases, National Institute for Viral Disease Control and Prevention, China CDC, Beijing, China; ^4^ NHC Key Laboratory of Medical Virology and Viral Diseases, National Institute for Viral Disease Control and Prevention, China CDC, Beijing, China

**Keywords:** respiratory syncytial virus, fumarprotocetraric acid, geraniin, antiviral, natural products

## Abstract

**Introduction:**

Respiratory syncytial virus (RSV) remains a major international public health concern. However, disease treatment is limited to preventive care with monoclonal antibodies and supportive care. In this study, natural products were screened to identify novel anti-RSV inhibitors.

**Methods:**

The antiviral effect of 320 compounds on RSV in HEp-2 cells was tested using a Cytopathic effect (CPE) inhibition assay. The antiviral effect of fumarprotocetraric acid (FUM) and geraniin (GE) were confirmed by Real-time reverse transcription quantitative PCR (Real-time RT-PCR), plaque reduction test, immunofluorescence assay, and Western blot analysis. Real-time PCR was used to detect inflammatory factor expression. ATP assay and JC-1 stain were used to evaluate mitochondrial protection function. The experiment of administration time was used to determine the stages in the RSV life cycle inhibited by FUM and GE. Human metapneumovirus (HMPV) and human rhinovirus (HRV) were used to evaluate the antiviral activities of other respiratory viruses of FUM and GE. Finally, Air-liquid interface human airway epithelium (ALI-HAE) cells were used to evaluate the antiviral effect and mechanism of FUM and GE to RSV.

**Results:**

The results showed that FUM and GE can inhibit the replication of RSV in multiple-cell models. Both compounds could dose-dependent inhibit the viral load, RSV nucleic acids level, and RSV-F protein level. Besides, FUM and GE showed good anti-inflammatory activity, mitochondrial protection, and antiviral activity to HMPV and HRV. Meanwhile, our result indicated that FUM and GE can inhibit RSV replication in ALI-HAE cells.

**Conclusions:**

FUM and GE were identified as new inhibitors of RSV infection. At the same time, FUM and GE have anti-inflammatory activity, mitochondrial protection function, and broad-spectrum antiviral activity. These results provide evidence that FUM and GE are potential candidates for the development of novel anti-RSV drugs.

## Introduction

The outbreak of the COVID-19 pandemic has caused a huge consequence on human health and the world economy. Although a variety of drugs have been marketed for COVID-19, drug development against the virus still faces major challenges. It is important to strengthen the research and development of novel antiviral drugs to overcome the emergence or reemergence of viral outbreaks.

Respiratory syncytial virus (RSV) is the second leading cause of death after malaria for infants in the world (Lozano et al., 2012). Nearly all infants have been infected with RSV at least once by the age of two years (Mazur et al., 2015). There were approximately 33 million cases of RSV-associated Acute lower respiratory infection (ALRI) in children under 5 years of age globally in 2019, and approximately 3.6 million infected children required hospitalization (Li et al., 2022b). RSV is also a major cause of acute respiratory illness in the elderly and immune-compromised individuals. Since naturally acquired immunity cannot produce persistent protection against RSV, re-infected can be seen throughout the lifetime (Johnson et al., 1962; Openshaw et al., 2017). There are only three licensed drugs for the treatment of RSV infection. Ribavirin (nucleoside analog), Palivizumab (humanized monoclonal antibody), and Nirsevimab (humanized monoclonal antibody). However, ribavirin is no longer recommended because of insufficient evidence of effectiveness, and Palivizumab is limited because of its high cost (The IMpact-RSV Study Group, 1998; Ralston et al., 2014). It is necessary to develop new anti-RSV drugs.

Considering the lack of drug candidates and the growing resistance to existing antiviral drugs, natural products may offer a promising source for identifying antiviral bioactive metabolites. Researchers have found many novel bioactive compounds like gnidicin, gniditrin, rutin, apigenin, and quercetin, which showed high efficacy in inhibiting several viruses such as HIV, SARS-CoV-2, HBV, and RSV (Sharma et al., 2023). One study found that three phenolic compounds of Sargassum fusiforme were active components against RSV infection *in vitro* (Chathuranga et al., 2021). Moreover, a series of flavonoids were found to have an antiviral effect against RSV from the seeds of Hovenia dulcis Thunb (Xu et al., 2020).

In the present study, we tested 320 natural products to find inhibitors of RSV infection, and fumarprotocetraric acid (FUM) and geraniin (GE) showed potent antiviral effects against RSV infection in multiple cell models, besides, FUM and GE have anti-inflammatory activity, mitochondrial protection function, and broad-spectrum antiviral activity. These results suggested that FUM and GE are potential candidates for novel anti-RSV drug research.

## Materials and methods

### Reagent preparation

320 natural products were obtained from the Institute of Materia Medica of the Chinese Academy of Medical Sciences (Beijing, China), and Ribavirin (RBV) was obtained from Meiruier Biochemical Technology Co., Ltd. (Cat no. 36791-04-5, Shanghai, China). FUM was obtained from ShanghaiyuanyeBio-TechnologyCo., Ltd. (Cat no. B74671, Shanghai, China), and GE was obtained from TargetMol Chemicals Inc. (Cat no. B74671, USA). All compounds were dissolved in Dimethyl sulfoxide (99.5%, Cat no. D5879, Sigma, USA) to a 10 mg/mL concentration for use as stock solutions.

### Cell culture and virus infection

The HEp-2 cells (human epidermoid cancer cells), the A549 cells (human lung carcinoma cell line), the Vero cells (African green monkey kidney cells), and the RSV Long strain (A) and 9320 strain (B) were all kindly donated by the National Institute of Viral Disease Control and Prevention, China CDC. HEp-2 cells, A549 and Vero cells were maintained in Dulbecco’s Modified Eagle’s Medium (DMEM) (Cat no. C11995500BT, Invitrogen, USA) supplemented with 10% fetal bovine serum (FBS) (Cat no. 10099141c, Gibco, Australia) and 1% HEPES (Cat no. 15630-080, Gibco, USA) at 37 °C with a 5% CO_2_ environment. The RSV Long strain and 9320 strain were propagated on confluent HEp-2 cells with virus maintenance medium (DMEM + 2% FBS) at 37 °C with a 5% CO_2_ environment, and the virus titer was determined by plaque assay.

### Cytotoxicity assay

HEp-2 cells were seeded into 96-well plates (3×10^4^cells/well) and incubated for 24 h. Cells were treated with indicated concentrations of compounds. At 48h post-treatment, the crystal violet was added to cells at room temperature for 30min, then the cells were rinsed, and absorbance was measured at a wavelength of 570 nm using a microplate reader to calculate 50% cytotoxic concentration (CC_50_) using GraphPad Prism 9.0 software.

### IC_50_ detection assay

The antiviral activities of the test samples were measured using the IC_50_ detection assay. HEp-2 cells were seeded into 96-well plates and incubated for 24h. Then the cells were infected with 100 TCID_50_ RSV Long strain or 9320 strain for 2h, and the mock group cells were treated with an inactivated virus (incubated for 30min at 56°C). The 8 serial dilutions of each compound were added to the cells after the virus adsorption. The cell viability was detected at 48h post-treatment via crystal violet stain (as above) to calculate the 50% inhibitory concentration (IC_50_) using GraphPad Prism 9.0 software.

### Real-time reverse transcription PCR analysis

Total RNA was extracted from the cell culture supernatant using Magnetic Bead Methods Nucleic Acid Extraction Kit (Cat no. B200, Zybio Inc. China). The mRNA levels of the RSV-M gene were identified using a One-step RT-PCR Kit (Cat no. AM1005, Ambion, USA) according to the manufacturer’s protocols, forward primer (5′-GGCAAATATGGAAACATACGTGAA-3′), reverse primer (5′-TCTTTTTCTAGGACATTGTAYTGAACAG-3′), probe (FAM-CTGTGTATGTGGAGCCTTCGTGAAGCT-TAMRA) were used as previously described (Kodani et al., 2011). Viral gene copies were determined with the standard curve generated as described previously (Xue et al., 2011). In simple terms, the 84bp amplicon for the control was purified, and cloned into a pGEM-T easy vector. The recombinant plasmids were extracted and singly digested, and the RNA internal control was synthesized from linearized RSV-M/pGEM-T template using the RiboMAX™ Large Scale RNA Production Systems-T7 kit (Cat no. P1300, Promega, USA) according to the manufacturer’s protocols, RNA transcripts were purified with the RNeasy Mini Kit (Cat no. 74104, QIAGEN, Germany), and stored at -80°C. A standard curve was generated based on a dilution series of the RNA internal control (1×10^-9^-1× 10^−1^copies/μl).

### Plaque reduction test

Viral titer was assessed via True Blue substrate-stained plaque assay. Briefly, the cell culture supernatant was ten-fold serial diluted and added to HEp-2 cells in 96 well plates. After incubation for 2 h, the medium was substituted with a maintenance medium with 1.5% carboxymethyl cellulose sodium. The cells were further cultured for 48h. After fixation, the cells were stained with mouse anti-RSV F protein monoclonal antibody (Cat no. ab43812, Abcam, UK, 1:300 dilution), followed by Goat Anti-Mouse IgG H&L (HRP) (Cat no. ZB-5305, Beijing Zhongshanjinqiao Technology Co., LTD, China, 1:1000 dilution). Plaques were developed using TrueBlue substrate (Cat nos. 5510-0050, KPL, USA), and the number of plaques was counted with a microscope.

### Immunofluorescence assay

HEp-2 cells were seeded into 96-well plates and incubated for 24h. Then the cells were infected with 100 TCID_50_ RSV Long strain for 2h. The 3 serial dilutions of each compound were added to the cells after the virus adsorption. The cell was fixed at 24h post-infection, and stained with mouse anti-RSV F protein monoclonal antibody, followed by Goat Anti-Mouse IgG DyLight™ 488 (Cat no. 35503, Thermo Scientific, USA, 1:1000 dilution). Finally, nuclei were counterstained with DAPI (Cat no. D9542, Sigma, USA), and images were taken with a fluorescence microscope.

### Western blot analysis

Whole extracts from cells grown in 24-well plates for 48h were loaded on 12% sodium dodecyl sulphate-polyacrylamide gel electrophoresis (SDS-PAGE) and transferred onto Nitrocellulose Membrane (NC) membranes. Membranes were blocked in 5% unfitted milk for 2h at room temperature and then incubated with mouse anti-RSV F protein monoclonal antibody at 4 °C overnight. After washing, membranes were incubated with Goat Anti-Mouse IgG H&L (HRP) for 2 h at room temperature. The signal densities were measured by an electrochemiluminescence system. GAPDH (Cat no. 10494-1-AP, Proteintech, China) was used as internal reference protein control. The RSV-F protein level was evaluated through a semi-quantitative analysis.

### Real-time PCR analysis

To determine cytokine level, RNA was extracted with whole extracts using FreeZol Reagent (Cat no. R711, Vazyme, China), and cDNA synthesis was performed using the HiScript II 1st Strand cDNA Synthesis Kit (Cat no. R211, Vazyme, China). Next, Real-time PCR was performed using the AceQ Universal SYBR qPCR Master Mix (Cat no. Q511, Vazyme, China). The expression of cytokine mRNA was analyzed using the 2^−ΔΔCt^ formula and expressed as fold induction, GAPDH was used as an internal control. The Real-time PCR primer sequences are shown in the **Supplementary Table S1**.

### ATP level detection

HEp-2 cells were seeded into 96-well plates and incubated for 24h. Then the cells were infected with 100 TCID_50_ RSV Long strain for 2h. The 3 serial dilutions of each compound were added to the cells after the virus adsorption. Following the manufacturer’s protocols, the ATP level was detected at 48h post-treatment via CellTiter-Glo Luminescent Cell Viability Assay Reagent (Cat no. G7570, Promega, USA).

### Mitochondrial membrane potential analysis

The values of MMP were determined with the JC-1Assay Kit (Cat no. C2003S, Beyotime, China) according to procedures. Briefly, the treated cells were collected and washed twice with PBS, then incubated with JC-1 (10 mM) for 30 min at 37 °C. After incubation, cells were washed twice with PBS and then analyzed with flow cytometry.

### Analysis of administration time

HEp-2 cells were seeded into 96-well plates and incubated for 24h. The cells were infected with RSV of about 100TCID_50_. In the pre-treatment group, drugs containing different dilutions were added 2h before infection. In the simultaneous treatment group, the drugs were added simultaneously as the virus infection progressed (2h). In the post-infection administration group, the drugs were added post-virus infection (46h). The cell viability was detected at 48h post-treatment via crystal violet stain to calculate the IC_50_ using GraphPad Prism 9.0 software.

### Inhibitory effect of FUM and GE on human metapneumovirus and human rhinovirus

HMPV strain, HRV strain, H1-Hela cells (human Cervical cancer cells), and LLC-MK2 cells (Rhesus Monkey Kidney Epithelial Cells) were kindly donated by the National Institute of Viral Disease Control and Prevention, China CDC. H1-Hela cells and LLC-MK2 cells were maintained in DMEM supplemented with 10% FBS, and 1% HEPES at 37 °C with a 5% CO_2_ environment. The antiviral activities of FUM and GE on HMPV and HRV were measured using Real-time RT-PCR. For HMPV, LLC-MK2 cells were seeded into 24-well plates and incubated for 24h. Then the cells were infected with HMPV at MOI=0.01 for 2h. The 3 serial dilutions of each compound were added to the cells after the virus adsorption. At 48h post-infection, the mRNA levels of the HMPV-N gene in the cell culture supernatant were identified using a One-step RT-PCR Kit according to the manufacturer’s protocols, forward primer (N-F:5’-CATATAAGCATGCTATATTAAAAGAGTCTC-3’), reverse primer (N-R:5’-CCTATTTCTGCAGCATATTTGTAATCAG-3’), probe (FAM-TGYAATGATGAGGGTGTCACTGCGGTTG-TAMRA) were used as previously described (Maertzdorf et al., 2004). Viral gene copies were determined with the standard curve. In the same way, the mRNA levels of HRV-5’UTR in H1-Hela cells were identified using the Real-time RT-PCR (as above), forward primer (5’UTR-F:5’-TGGACAGGGTGTGAAGAGC-3’), reverse primer (5’UTR-R:5’-CAAAGTAGTCGGTCCCATCC-3’), probe (FAM-TCCTCCGGCCCCTGAATG-TAMRA) were used as previously described (Maertzdorf et al., 2004; McLeish et al., 2012).

### HAE cell culture

Primary HAE cells were kindly donated by the National Institute of Viral Disease Control and Prevention, China CDC. HAE cells are cultured as described previously (Chen et al., 2019). Briefly, HAE cells were cultured on a type I/III collagen (Cat no. 04902, Stemcell, Canada)-covered 6-well plate with Bronchial Epithelial Cell Medium (Cat no. sc-3211, BMCM, ScienCell, USA) containing additives in a 37 °C incubator with 5% CO_2_. Once the cells reached 80%–90% confluence, the cells were digested and plated at a density of 3 × 10^5^ cells/well on type IV collagen (Cat no. 5022, Advanced BioMatrix, USA)-coated Transwell plates (Cat no. 3470, Costar, USA). The culture medium for both the apical and basolateral sides was renewed every other day. Once the cells formed tight junctions, the medium was changed to HAE medium (BMCM + DMEM + additives) and cultured for another 5 days, then HAE cells were cultured at an air-liquid interface (ALI) for 4–6 weeks to form a pseudostratified structure.

### Anti-RSV activity of FUM and GE on ALI-HAE cells

The cytotoxicity of FUM and GE to HAE cells was determined by Lactate dehydrogenase (LDH) assay. Well-differentiated HAE cells were treated with FUM and GE. The supernatant was collected every other day by adding a 200μl medium to the apical side for 1h, and the release of LDH was detected with Cytotoxicity Det.Kit (Cat no. 04744926001, Roche, Switzerland). Then, HAE cells were infected with RSV Long strain (MOI=0.01) for 2h. FUM and GE were added to the basolateral chamber. The release of RSV nucleic acid was collected every other day, and virus gene copies were detected by Real-time RT-PCR (as above).

### Transcriptome analysis of RSV resistance of FUM and GE on ALI-HAE cells

Uninfected cells, infected cells, and treated HAE cells were lysed on the fifth day of administration after infection, and RNA was extracted using RNeasy Mini Kit. The extracted RNA was sent to BGI Shenzhen for transcriptome library construction and RNA sequencing using the DNBSEQ platform (double-ended sequencing, 150bp). Differentially expressed genes (DEGs) were enriched according to the Phyper function of the negative binomial distribution, including GO enrichment analysis and KEGG pathway analysis. Significant differences were defined with *Q* value<0.05 and Log (Fold Change) >1. Real-time PCR (as above) was performed to verify the result of DEGs, and GAPDH was used as an internal control. The primer sequences are shown in the **Supplementary Table S2**.

### Statistical analysis

The data were described as mean ± SEM, and are representative of at least three independent experiments. Graphs and all Statistical analysis were performed using GraphPad Prism 9.0 software (San Diego, USA). Differences between the vehicle group and untreated or treated groups were analyzed by one-way analysis of variance (ANOVA), and *p* < 0.05 was considered statistically significant.

## Results

### Antiviral effects of FUM and GE

**Figure 1:**
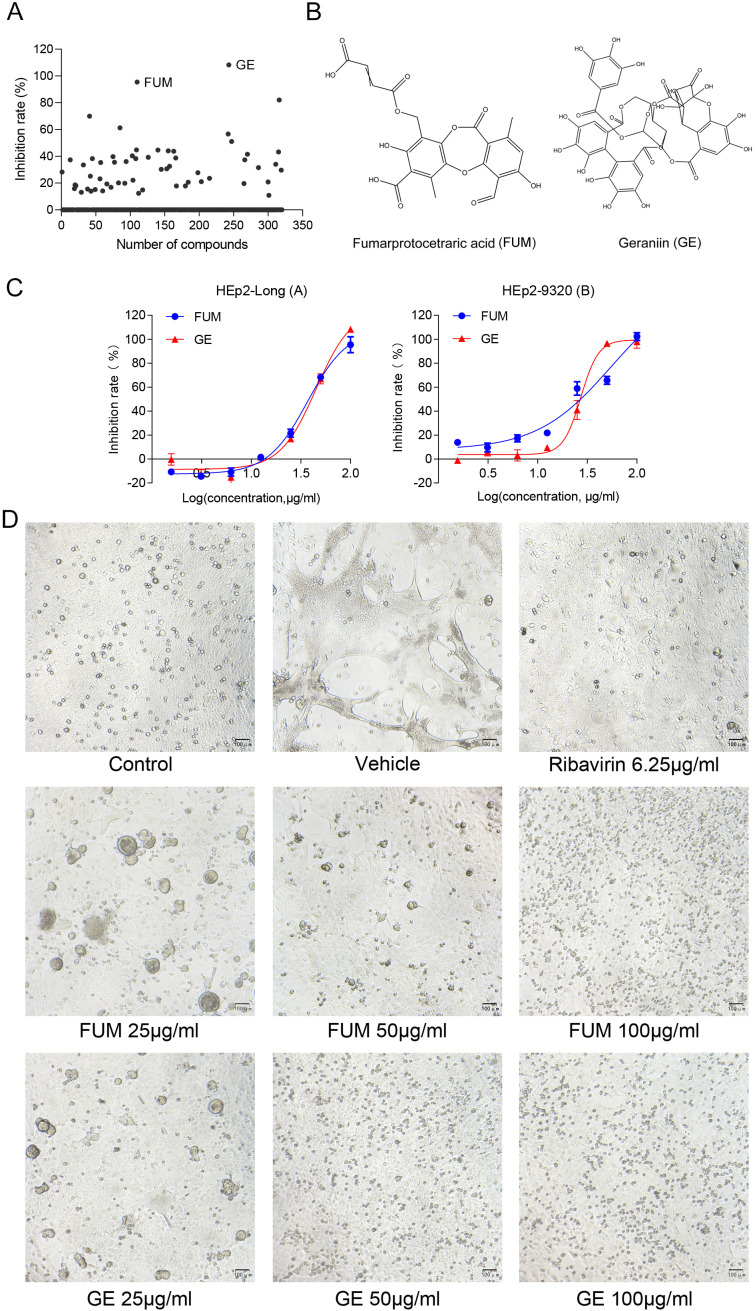
Antiviral activity of fumarprotocetraric acid (FUM) and geraniin (GE) against RSV in cell-based assays. **(A)** Inhibit rate of 320 nature products. **(B)** Chemical structures of FUM and GE. **(C)** Concentration-inhibition response curve of FUM and GE against different RSV strains in HEp-2 cells. **(D)** The CPE images of FUM and GE against RSV Long strain in HEp-2 cells were obtained with the microscope (100×, scale bar = 100 μm).

To assess the effects of natural products on RSV infection, HEp-2 cells were infected with RSV Long strain (100 TCID_50_) and treated with indicated concentrations of different compounds. After 48 h, the Cytopathic effect (CPE) was determined with the crystal violet staining method, and IC_50_ was calculated using the spectrophotometric data. Among the 320 compounds, we found that FUM and GE had the best antiviral activity within the safe dose range, IC_50_ is 37.07 μg/ml and 45.03 μg/ml respectively (**Figures 1A–C**, **Table 1**). Both compounds showed an inhibiting effect on RSV A/B strains and were equally effective on HEp-2 cells. In addition, FUM and GE can inhibit typical syncytial formation caused by RSV (**Figure 1D**).

**Table 1:** *In vitro* antiviral activity of fumarprotocetraric acid (FUM) and geraniin (GE) against RSV A/B strains using the CPE reduction assay.

Virus	Compounds	CC_50_ (μg/ml)^a^	IC_50_ (μg/ml)^b^	SI^c^
Long strain (A)	FUM	>100	37.07	>2.70
	GE	>100	45.03	>2.22
9320 strain (B)	FUM	>100	36.6	>2.73
	GE	>100	92.2	>1.08

a CC_50_: median toxic concentration.

b IC_50_: 50% inhibitory concentration.

c SI: selectivity index, SI= CC_50_/EC_50_.

### Effect of FUM and GE on the virus replication in HEp-2 cells

**Figure 2:**
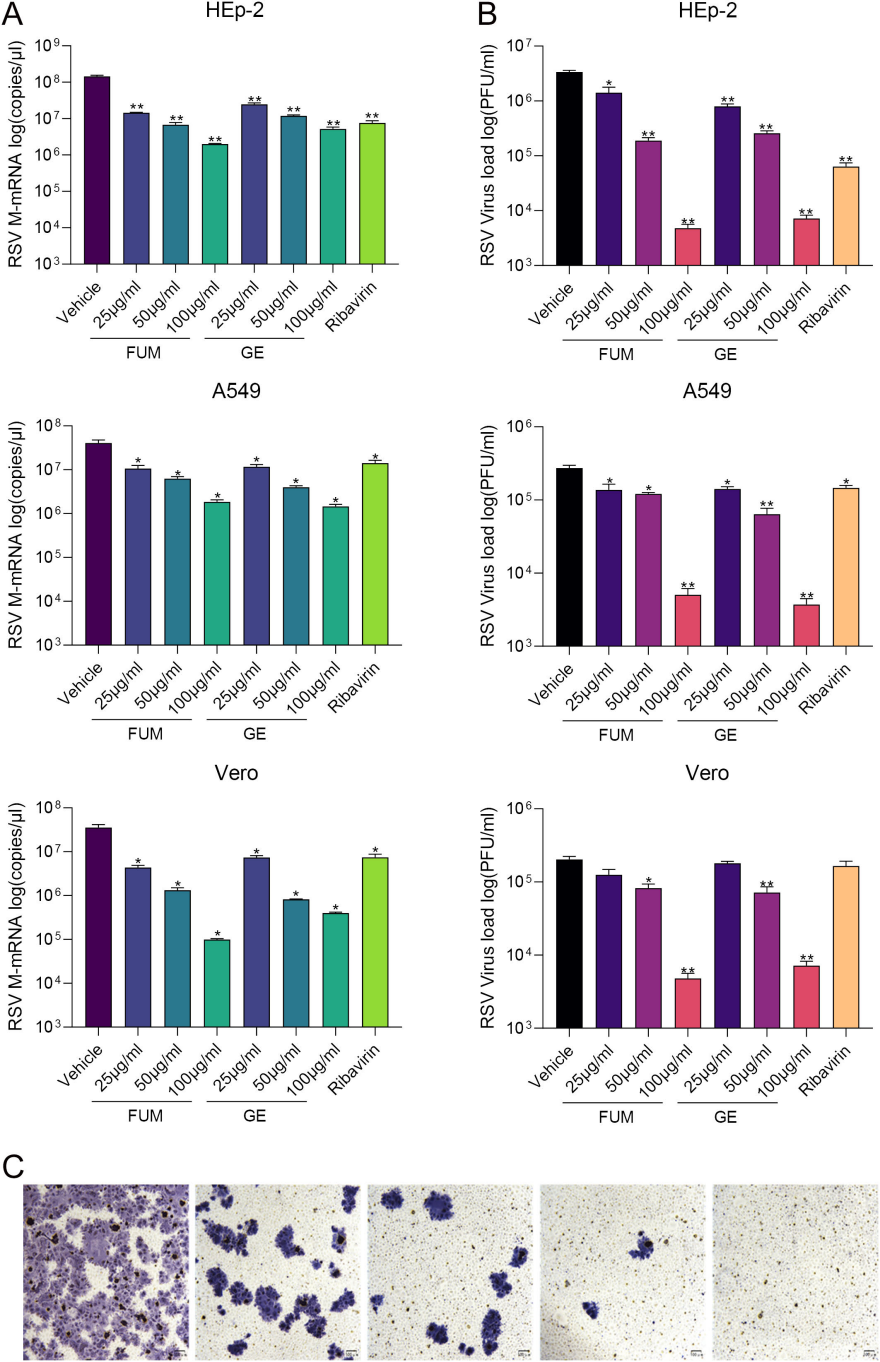
Fumarprotocetraric acid (FUM) and geraniin (GE) inhibited virus replication in RSV-infected HEp-2 cells, A549 cells, and Vero cells. **(A)** RSV-M gene level detected by Real-time RT-PCR. **(B)** RSV viral titer was measured via plaque assay. Ribavirin group, 6.25μg/ml. **(C)** Representative image of plaque immunostaining results in wells at different dilutions. Virus plaque was labelled with anti-RSV-F antibody and HRP-conjugated secondary antibodies and then visualized with TrueBlue substrate (100×, scale bar = 100 μm). Data are described as the mean ± SEM (n = 3). *p < 0.05 and **p < 0.01, vs. the vehicle group.

**Figure 3:**
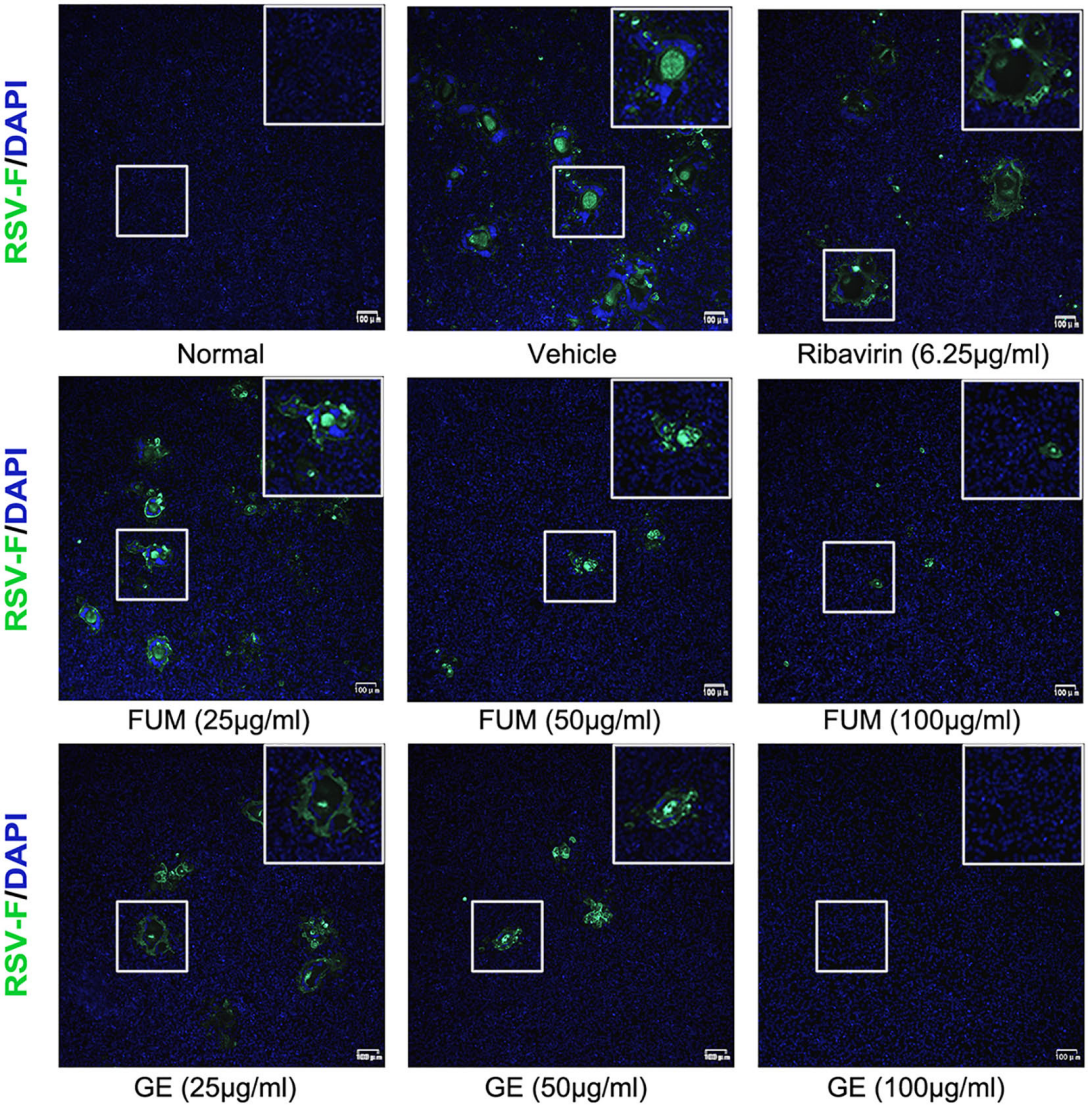
Immunofluorescence detection of RSV-F protein expression inhibited by fumarprotocetraric acid (FUM) and geraniin (GE) in HEp-2 cells.

**Figure 4:**
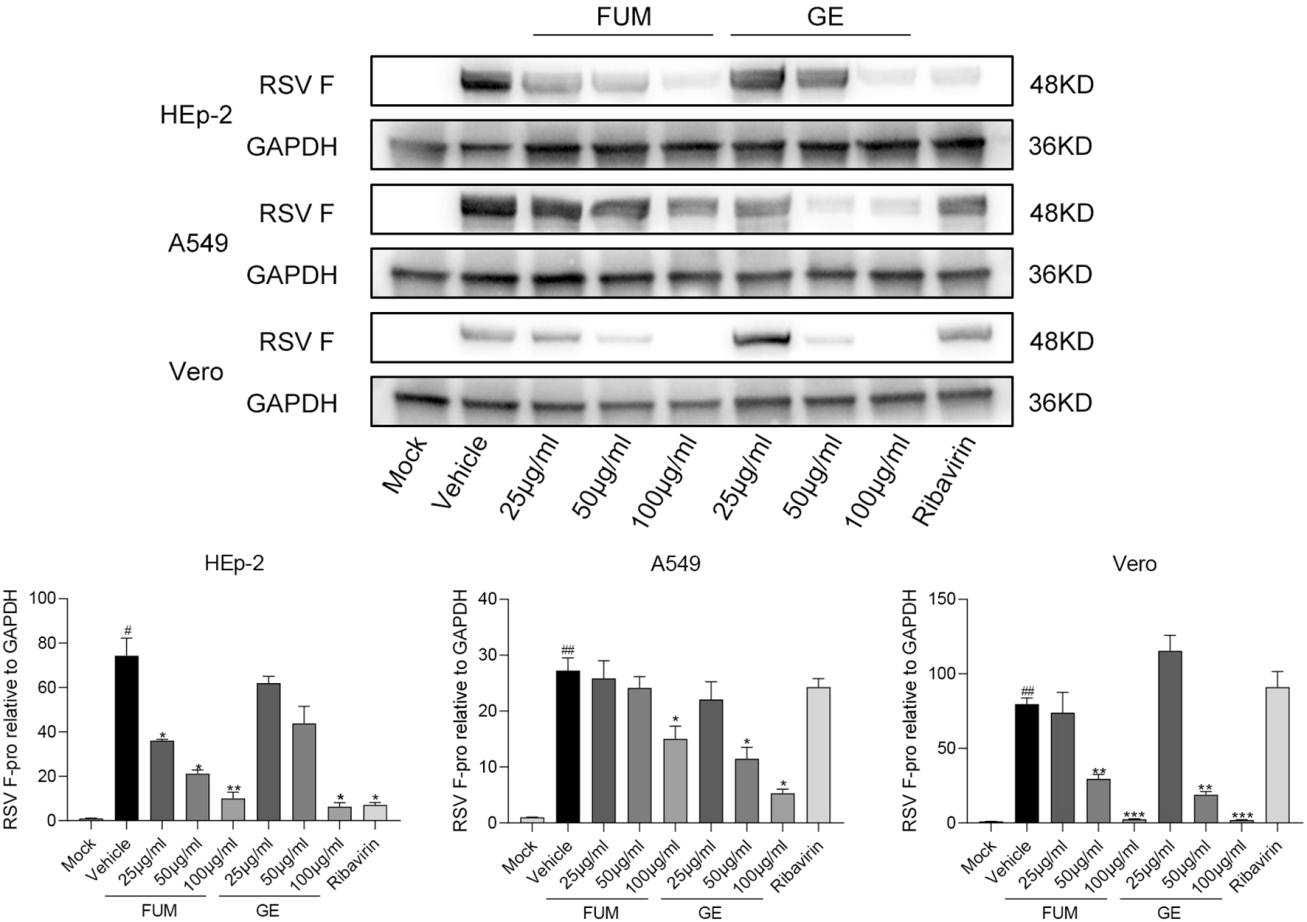
Fumarprotocetraric acid (FUM) and geraniin (GE) inhibited RSV-F protein expression assessed via Western blot in HEp-2 cells, A549 cells, and the Vero cells. Ribavirin group, 6.25μg/ml. Data are described as the mean ± SEM (n = 3). # *p* < 0.05 and ## *p* < 0.01, compared to the mock group. *p < 0.05, **p < 0.01, and ***p < 0.001 vs. the vehicle group.

To confirm the anti-RSV activity of FUM and GE, HEp-2 cells, A549 cells, and Vero cells were infected with RSV Long strains (MOI = 0.01) and treated with different concentrations of FUM and GE for 48 h. As shown in **Figure 2**, compared with the vehicle group, the nucleic acid levels of the RSV-M gene detected by Real-time RT-PCR and the viral titer of the treated group detected by plaque reduction assay was significantly decreased by FUM and GE in three cells. Immunofluorescence results also indicated that FUM and GE can inhibit RSV replication and reduce syncytium formation in HEp-2 cells (**Figure 3**). In addition, the Western blot results showed that FUM and GE can dose-dependent decrease RSV-F protein levels in three cells (**Figure 4**). Finally, to determine the antiviral effect of FUM and GE in different TCID_50_, we performed a nucleic acids test. The results proved that FUM and GE can inhibit the RSV-M gene in 0.01MOI, 0.001MOI, and 0.0001MOI (**Supplementary Figure S1**). Taken together, the results indicated that FUM and GE could play an antiviral role in different cell models.

### Inflammatory factor expression regulation by FUM and GE

**Figure 5:**
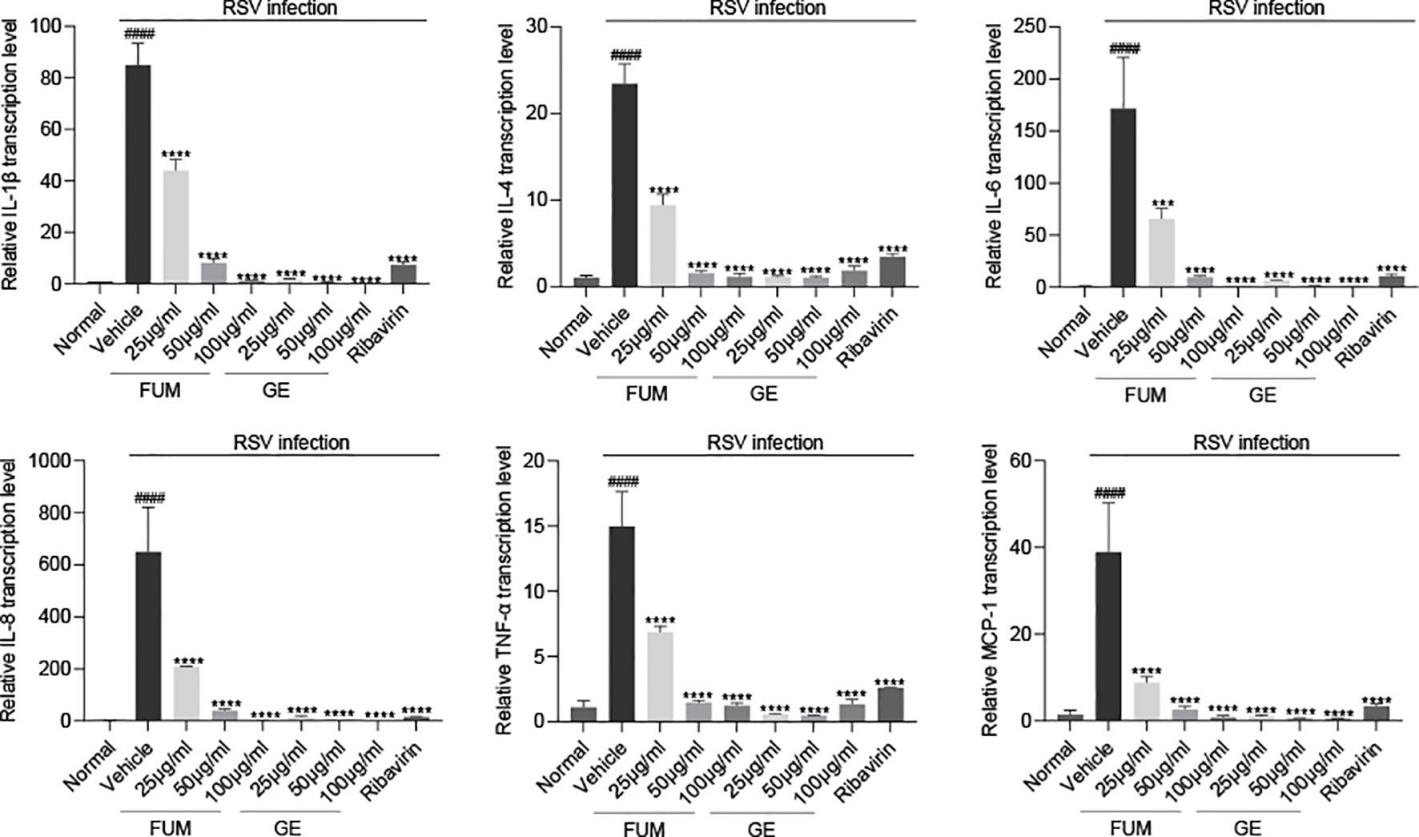
Effect of fumarprotocetraric acid (FUM) and geraniin (GE) on cytokine production in RSV-infected cells. The transcription levels of mRNA of IL-1β, IL-4, IL-6, IL-8, TNF-α, and MCP-1 in HEp-2 cells were detected by Real-time PCR. Ribavirin group, 6.25μg/ml. Data are described as the mean ± SEM (n = 3). #### *p* < 0.0001, compared to the mock group. ***p < 0.001 and ****p < 0.0001 vs. the vehicle group.

The pathology caused by RSV is mainly caused by the injury of the lung epithelium caused by excessive inflammation (Bohmwald et al., 2019). The cytokines and chemokines levels induced by RSV Long strain were detected with Real-time PCR to assess the anti-inflammatory effect of FUM and GE. As shown in **Figure 5**. Both FUM and GE can significantly reduce the transcription levels of mRNA of IL-1β, IL-4, IL-6, IL-8, TNF-α, and MCP-1 induced by RSV infection, suggesting that FUM and GE have anti-inflammatory effects.

### Effects of FUM and GE on ATP and MMP

**Figure 6:**
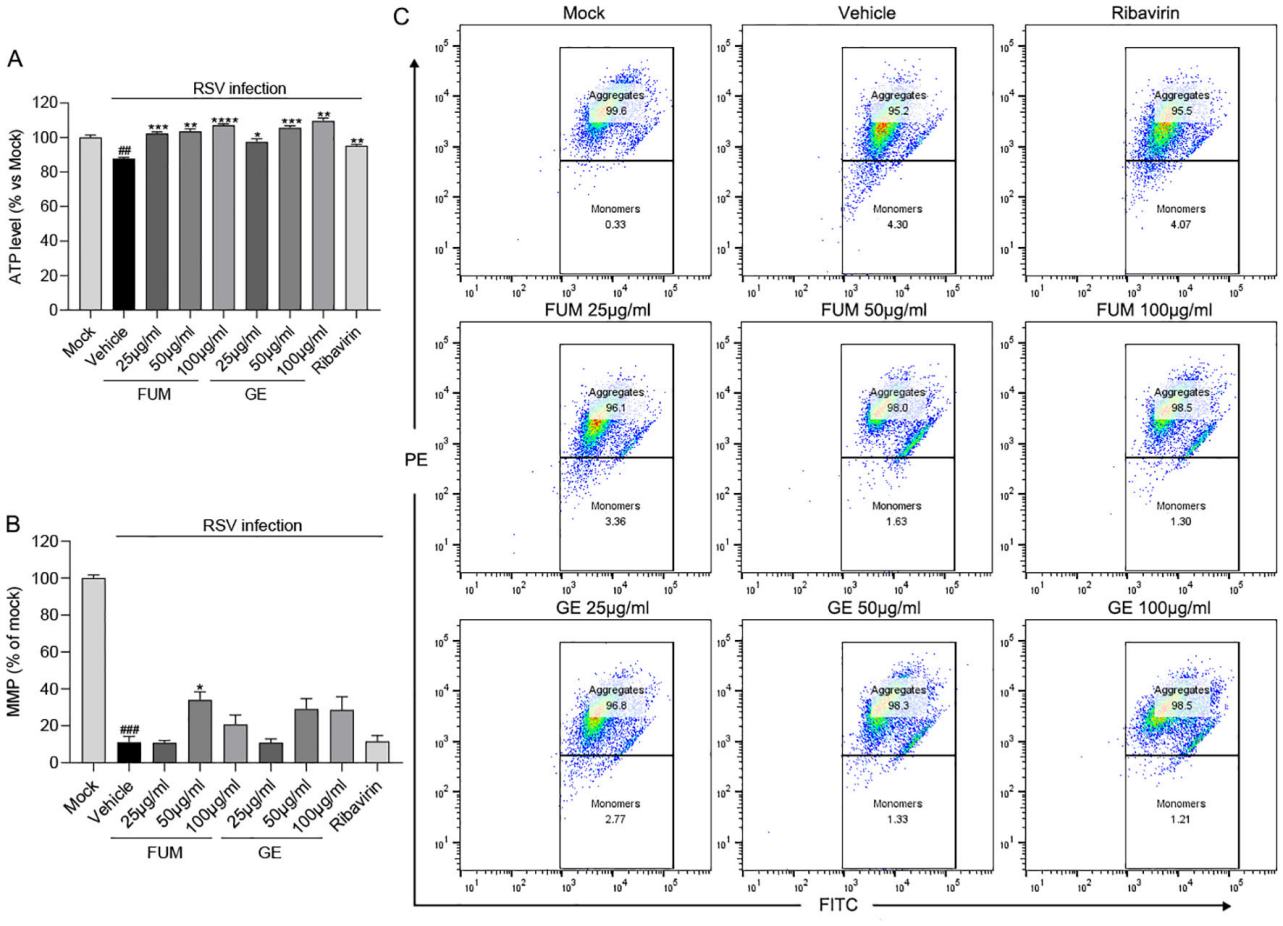
The effects of fumarprotocetraric acid (FUM) and geraniin (GE) on mitochondrial function in RSV-infected cells. **(A)** The effects of FUM and GE on cell ATP level assessed by CellTiter-Glo Luminescent Cell Viability Assay in HEp-2 cells. **(B, C)** Mitochondrial Membrane Potential (MMP) Analysis of infected cells after FUM and GE treatment for 48h by flow cytometry analysis. Ribavirin: 6.25 μg/ml. Data are described as the mean ± SEM (n = 3). ## *p*< 0.01, and ### *p*< 0.001, compared to the mock group. *p < 0.05, **p< 0.01, ***p< 0.001, and ****p< 0.0001 vs. the vehicle group.

We next examined the effects of FUM and GE on mitochondrial function. As reported, RSV infection could decrease the mitochondrial respiration rate (Grunwell et al., 2018), our results showed that the level of ATP and the value of MMP in infected cells was significantly decreased (*p*<0.01 and *p*<0.001, respectively), while FUM and GE could increase the concentration of ATP and MMP value (**Figure 6**). The result suggests that FUM and GE may have the ability to protect mitochondrial function.

### Analysis of administration time of FUM and GE

**Figure 7:**
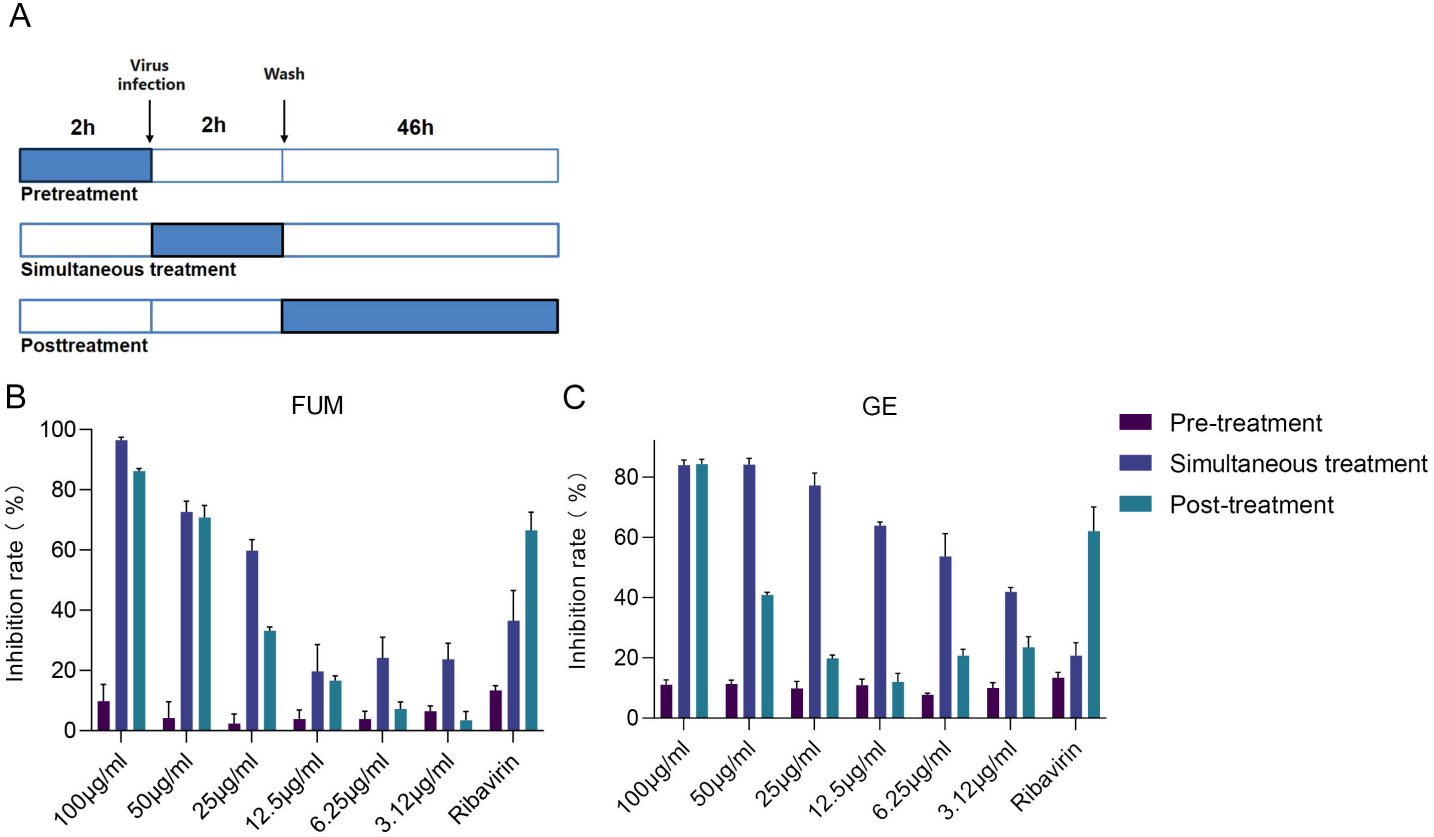
Time of addition analysis of fumarprotocetraric acid (FUM) and geraniin (GE). **(A)** Schematic representation of the time-of-addition analysis. **(B)** The dose-effect curve under three administration times of FUM. **(C)** The dose-effect curve under three administration times of GE. Ribavirin: 6.25 μg/ml. Data are described as the mean ± SEM (n = 3).

To find out which stage FUM and GE play an antiviral role, we conducted an administration time analysis. The results showed that both FUM and GE could not inhibit RSV in the pre-treatment group (compounds treatment 2h before infection). For FUM, there was no significant difference in the antiviral effect between simultaneous administration (compounds treatment 2h simultaneously during virus infection) and post-infection administration (compounds treatment 46h post-infection), the IC_50_ values of 27.48μg/ml and 33.08μg/ml, respectively. For GE, the antiviral effect of simultaneous administration is better than that of post-infection administration. The IC_50_ values were 8.5μg/ml and 55.83μg/ml, respectively, suggesting that the antiviral effect of GE mainly affects the entry stage of the virus life progress (**Figure 7**, **Table 2**).

**Table 2:** Time of addition analysis of fumarprotocetraric acid (FUM) and geraniin (GE).

Virus	Compounds	IC_50_ (μg/ml) ^a^
pre-treatment group	FUM	–
	GE	–
simultaneous-treatment group	FUM	27.48
	GE	55.83
post-infection-treatment group	FUM	33.08
	GE	8.5

a IC_50_: 50% inhibitory concentration.

### Inhibitory effects of FUM and GE on other respiratory viruses

**Figure 8:**
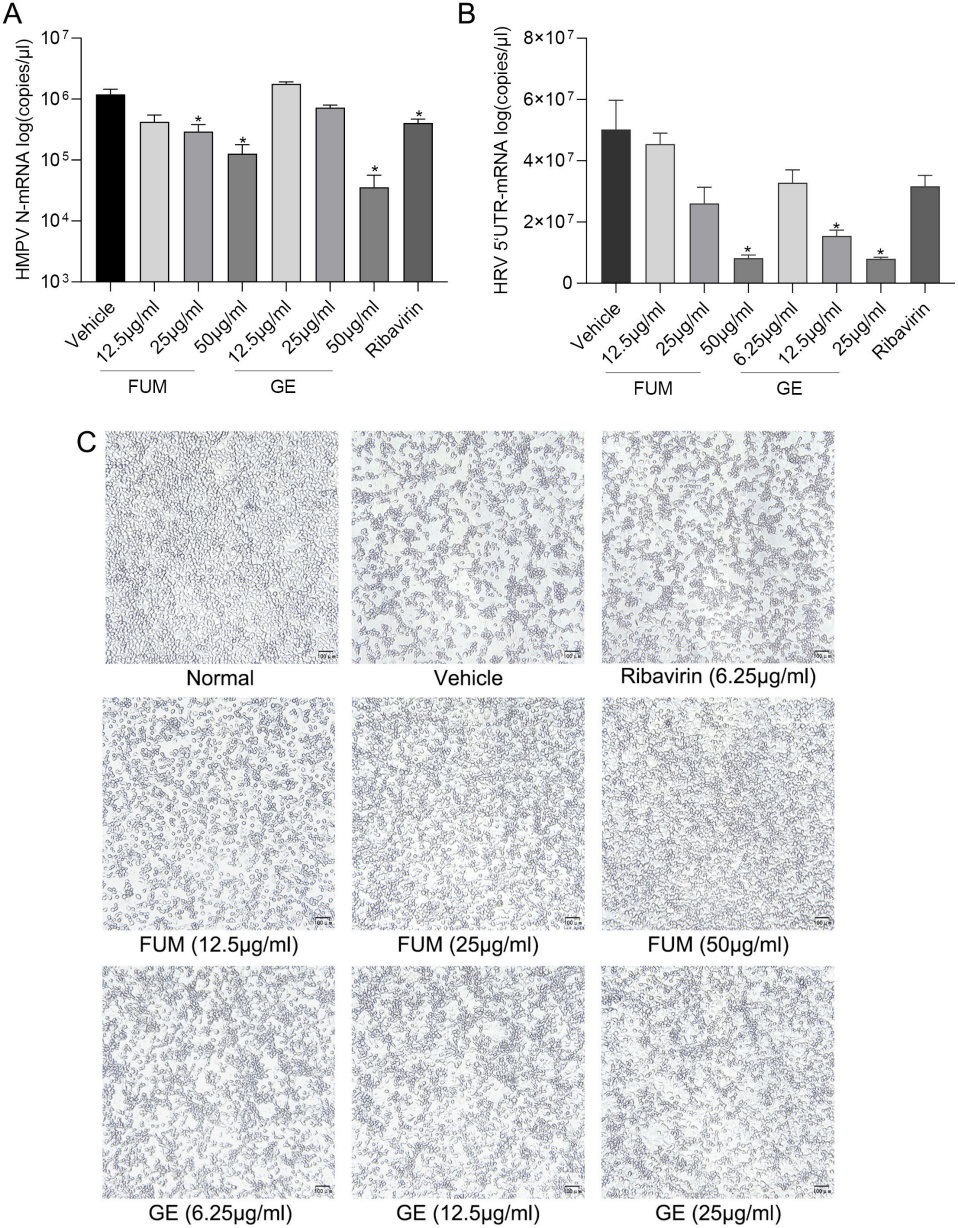
Fumarprotocetraric acid (FUM) and geraniin (GE) inhibited respiratory virus in infected cell models. **(A)** The inhibiting effect of FUM and GE to HMPV-N gene nucleic acids level in LLC-MK2 detected by Real-time RT-PCR. **(B)** The inhibiting effect of FUM and GE to HRV-5’UTR nucleic acids level in H1-Hela detected by Real-time RT-PCR. **(C)** HRV CPE images were obtained with the microscope (100×, scale bar = 100 μm). Ribavirin group, 6.25μg/ml. Data are described as the mean ± SEM (n = 3). * *p* < 0.05 vs. the vehicle group.

To test whether FUM and GE can inhibit other respiratory virus infections, we evaluated the anti-HMPV activity of FUM and GE in the LLC-MK2 cell model, and the anti-HRV effects of FUM and GE on H1-Hela cells. HMPV and HRV are common viral pathogens responsible for the human respiratory tract infection. Real-time RT-PCR results showed that FUM and GE could dose-dependent inhibit both HMPV and HRV nucleic acid levels (**Figures 8A, B**). In addition, the CPE inhibition assay showed that FUM and GE could inhibit HRV CPE, the IC_50_ values were 10.5μg/ml and 3.73μg/ml, respectively (**Figure 8C**). These results suggest that FUM and GE have potential broad-spectrum antiviral activity.

### FUM and GE decrease the virus replication in ALI-HAE cells

**Figure 9:**
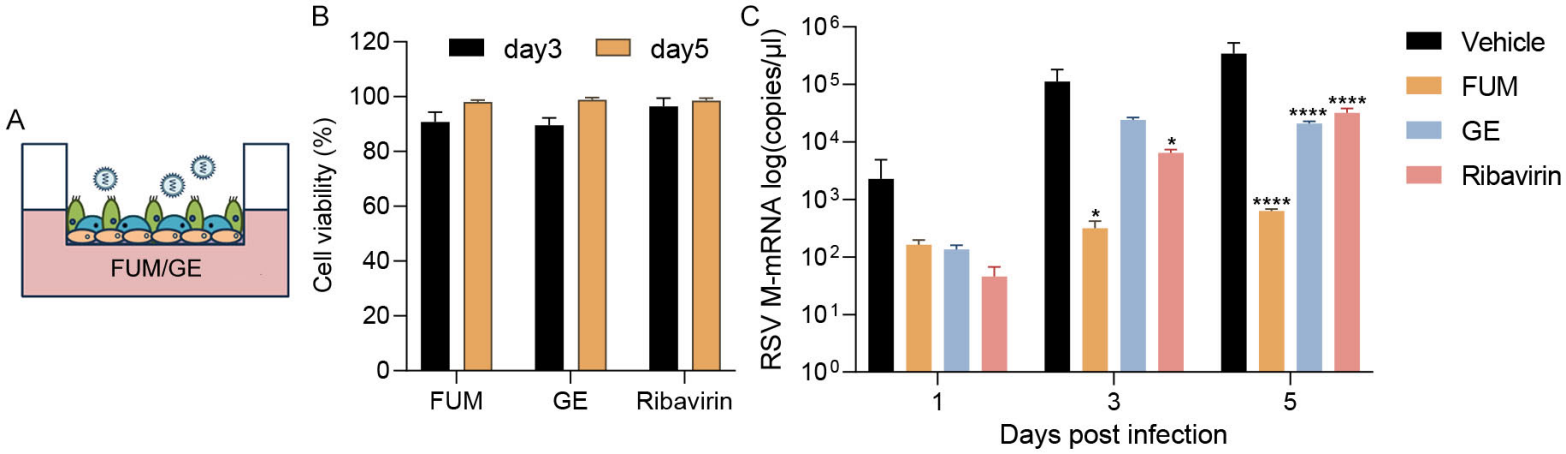
Effect of fumarprotocetraric acid (FUM) and geraniin (GE) on virus replication in RSV-infected human airway epithelium (HAE) cells. **(A)** HAE cells were infected with RSV Long strain (0.01MOI). FUM and GE were added to the basolateral chamber of the Transwells (FUM and GE, 100μg/ml; Ribavirin, 6.25μg/ml). **(B)** Cytotoxic effects of FUM and GE on HAE cells detected by LDH. **(C)** Real-time RT-PCR detected collected virus RSV-M gene levels from the apical side. Data are described as the mean ± SEM (n = 3). *p < 0.05 and ****p < 0.0001 vs. the vehicle group.

ALI-HAE cells can form a pseudostratified architecture including basal, ciliated, mucus-producing goblet cells, which could realistically simulate an RSV infection environment in the human body (Essaidi-Laziosi et al., 2018). We evaluated the antiviral efficacy of FUM and GE in ALI-HAE cells. After apical infection with RSV Long strain, adding the compounds to the basolateral chamber (**Figure 9A**). Real-time RT-PCR detected the release of RSV nucleic acids from the apical side. The results showed that both FUM and GE (100μg/ml) have no cytotoxicity to HAE (**Figure 9B**), and can significantly reduce the RSV-M gene nucleic acids levels in HAE cells (**Figure 9C**).

### RNA-seq analysis of FUM and GE on RSV-infected HAE cells

**Figure 10:**
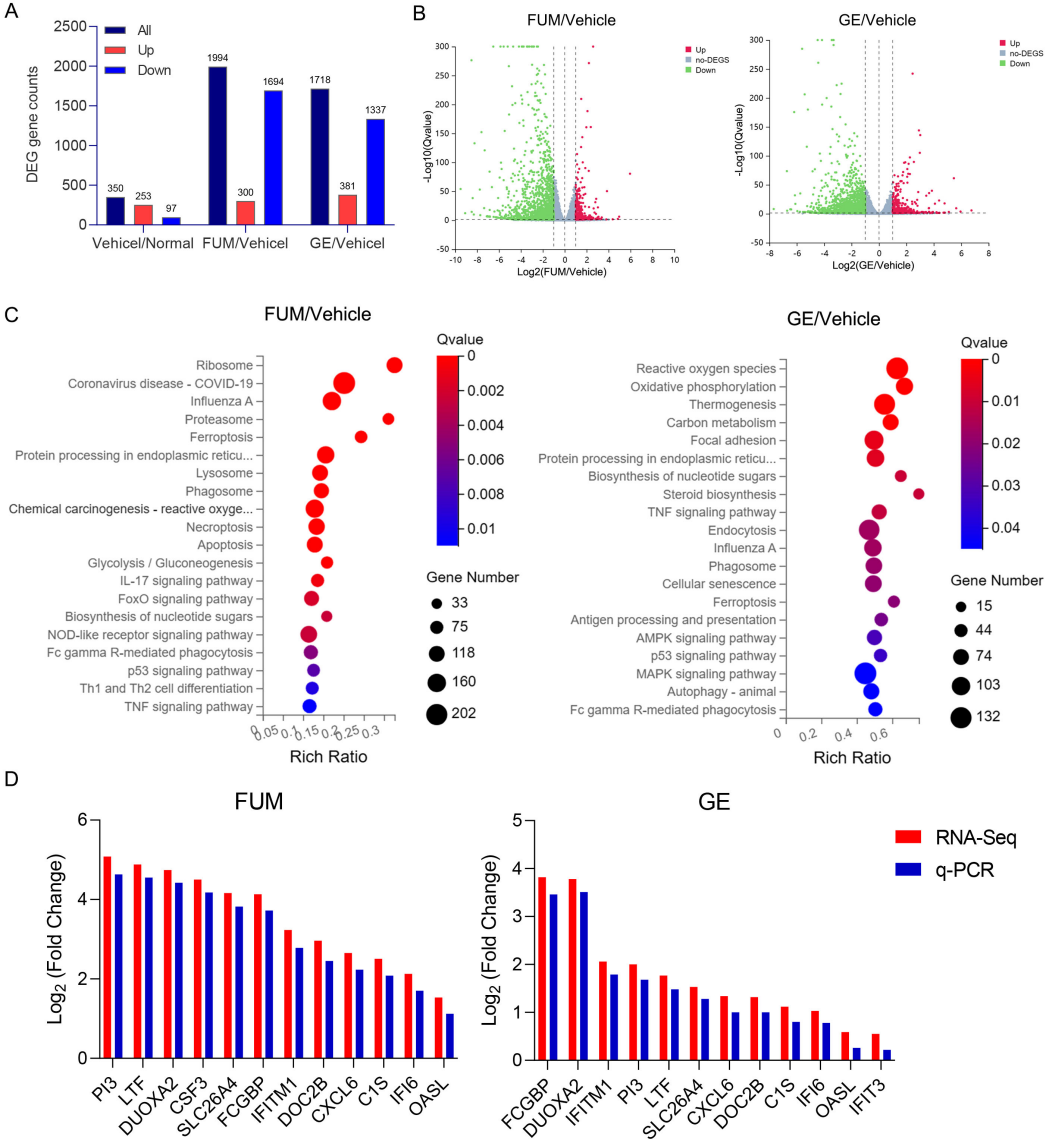
Effects of fumarprotocetraric acid (FUM) and geraniin (GE) on differentially expressed genes (DEGs) in RSV-infected human airway epithelium (HAE). **(A)** The number of DEGs between each group. **(B)** Volcano map shows up-regulated and down-regulated DEGs in each group (Green: down-regulated genes; Grey: no significant DEGs; Red: Up-regulated gene). **(C)** Bubble plot of KEGG enrichment of all DEGs. The bar plot: the length represents the number of genes, the abscissa is the ratio of the number of DEGs annotated on the KEGG term to the total number of DEGs, and the ordinate represents the KEGG term. **(D)** The Real-time PCR analysis of the DEGs mRNA expression in HAE cells.

To investigate the potential mechanism of anti-RSV action of FUM and GE, uninfected cells, infected cells, and treated HAE cells were lysed, and total RNA was extracted and sent to the company for RNA-Seq analysis. *Q* value<0.05 and Log (Fold Change) >1 was used to define DEGs. Analysis results showed that 253 genes were up-regulated and 97 genes were down-regulated in the virus group compared with the normal group. Compared with the virus group, there were 300 up-regulated genes and 1694 down-regulated genes in the FUM administration group. Compared with the virus group, 381 genes were up-regulated and 1337 genes were down-regulated in the GE administration group (**Figures 10A, B**). *Q* value<0.05 and Log (Fold Change) >1 criteria were used to analyze KEGG pathway enrichment analysis, and the results showed that the biological processes regulated by FUM and GE were mainly in the signal transduction process of infectious diseases, regulating cell growth, lipid metabolism, immune system function, and endocrine system function. FUM may regulate processes such as the ribosome, proteasome, lysosome, endocytosome, apoptosis, IL17, FoxO, and NOD-like receptor signaling pathways. GE may regulate reactive oxygen species, phosphorylation, thermogenesis, TNF, AMPK, P53, and MAPK signal pathway (**Figure 10C**). The expression of DEGs was verified by Real-time PCR. Compared with the vehicle group, the mRNA expression of FUM and GE treated group showed the same trend with RNA-seq (**Figure 10D**). The mRNA expression of PI3, LTF, FCGBP, DUOXA2, and IFITM1 play a crucial role in the anti-RSV action of both FUM and GE.

## Discussion

According to a literature study, 33.1% of worldwide pneumonia is caused by RSV (Mazur et al., 2023). Available antiviral drugs are mainly targeted at IFV, HIV, HSV, HBV, and HCV, and few antiviral drugs are targeted at RSV. With the emergence of new respiratory viruses around the world in recent years, such as influenza A (H1N1), SARS, MERS, and SARS-CoV-2, the development of new anti-RSV drugs can not only reduce the disease burden caused by RSV but also expand the library of antiviral compounds respond to emergence viral outbreaks.

Traditional medicinal plants have been used for thousands of years in folk medicines in the treatment of several diseases. Natural products from medicinal plants can provide a huge source for bioactive components screen. In this study, 320 natural products were screened to find inhibitors of RSV infection using a CPE reduction assay. Two active compounds (FUM and GE) were identified as hits with potent antiviral effects against RSV infection *in vitro*, and both agents did not show any cytotoxic effects in the HEp-2 cell under the concentration of 100 μg/ml. The IC_50_ of FUM and GE are 37.07 μg/ml and 45.03 μg/ml respectively. FUM is a compound found in many lichens that has been found to have antimicrobial activity and antioxidant activity (de Barros Alves et al., 2014; Yañez et al., 2023; Yilmaz et al., 2004). Moreover, the major metabolite of FUM (depsidone FUM) showed a neuroprotective potential (Fernández-Moriano et al., 2017), but the antiviral activity of FUM has not been reported. GE is a polyphenolic compound first isolated from Geranium thunbergia, which has various antiviral activities, such as anti-herpes simplex virus-1, enterovirus 71, dengue virus, and HBV (Abdul Ahmad et al., 2019; Liu et al., 2016; Siqueira et al., 2020; Yang et al., 2012). However, the antiviral activity of GE against RSV has not been reported.

Next, we confirmed the dose-dependent anti-RSV activity of FUM and GE in the three common cell lines via various methods. The results of Real-time RT-PCR, plaque assay, immunofluorescence assay, and Western Blot suggest that FUM and GE can significantly decrease the RSV nucleic acids level, viral titers, and protein levels. The high dose group (100μg/ml) of FUM and GE have better antiviral efficacy than the Ribavirin group (6.25μg/ml). These results demonstrated the potential antiviral activity of FUM and GE.

Excessive inflammation is the main reason for pulmonary pathology in RSV infection, and cytokines play an important role. High levels of inflammation in the airways can activate and attract large numbers of neutrophils, leading to tissue damage (Carvajal et al., 2019). Our finding indicated that FUM and GE can reduce inflammation, and decrease the the transcription levels of mRNA of IL-1β, IL-4, IL-6, IL-8, TNF-α, and MCP-1, indicating that both FUM and GE have anti-inflammatory effects. Meanwhile, RSV infection could lead to energy metabolism disorders and a reduction in ATP synthesis (Grunwell et al., 2018). Koshiba et al. found that a reduced mitochondrial membrane potential (ΔΨ(m)) correlated with the reduced antiviral response (Koshiba et al., 2011). Our results showed that FUM and GE can improve the ATP level and MMP value in RSV-infected cells, which could protect mitochondrial function damaged by virus infection.

Although the exact molecular target for FUM and GE is not known, administration time analysis can help to understand the general mode of action of the drug. The results showed that the FUM mainly played an antiviral role in early and post-infection administration, while GE mainly acts in the entry stage of the virus infection. Suggested that FUM and GE may play antiviral roles through different molecular mechanisms. FUM mainly affects the process of virus growth and replication, while GE may act as a small molecule entry inhibitor for RSV infection.

To investigate whether FUM and GE have the same effect on other respiratory viruses, we evaluated the anti-HMPV and anti-HRV activities. HMPV was first discovered in 2001 which is another member of the *Pneumovirida*e family, along with RSV (van den Hoogen et al., 2001). HMPV mainly causes upper and lower respiratory tract infections in children. HMPV can also occur in concentrated outbreaks among susceptible people, and even cause death in critically ill patients (Peña et al., 2019; Shafagati and Williams, 2018). Notably, one case report stated that three children co-infected with HMPV and SARS-CoV-2 were found to have died, indicating that HMPV may influence susceptibility and pathogenicity of SARS-CoV-2 (Hashemi et al., 2021). HRV is an unenveloped single-stranded RNA virus, belonging to the *Picornavirudae* family. HRV is an etiologic agent of the common cold, which is commonly found in all age groups. Researchers found that HRV is the most common cause of wheezing in children with asthma, and once asthma is established, HRV infection is a potent trigger for acute airway obstruction and exacerbations in children and adults (Jackson and Gern, 2022; Jartti et al., 2009). The real-time RT-PCR results indicated that FUM and GE could dose-dependent inhibit both HMPV and HRV nucleic acid levels, and both compounds could inhibit the CPE of HRV. These results suggest that the two drugs have potential broad-spectrum antiviral activity.

Apart from chimpanzees, animal models such as cotton rats, mice, and ferrets are only semi-permissive for RSV infection and the infection shows little or no clinical signs (Taylor, 2017). Compared to general cell lines and animal models, HAE cells are a better model for respiratory virus infection research, which can mimic ‘‘*in vivo*’’ physiological conditions. ALI-HAE cells have been used for the culture of difficult-to-isolate respiratory viruses, vaccine research, and drug evaluation (Komabayashi et al., 2021; Lee et al., 2023; Li et al., 2022a; Liu et al., 2020). The evaluation of FUM and GE in HAE cells showed better antiviral efficacy than the ribavirin group. RNA-Seq analysis of FUM and GE on RSV-infected HAE cells showed the potential mechanism of anti-RSV action of FUM and GE. The results of the KEGG enrichment analysis of FUM mainly focus on the signaling pathways of viral infection, protein synthesis, inflammatory response, and innate immune response. The regulatory processes of GE mainly focus on oxidative stress, thermogenesis, inflammatory response, and viral infection signaling pathways, which are consistent with the results of inflammatory factor assays and mitochondrial function tests. The results of DEGs identified by RNA-Seq analysis were verified by Real-time PCR.

Our study identified two anti-RSV compounds, FUM and GE. Which also have anti-inflammatory activity, mitochondrial protection function, and broad-spectrum antiviral activity. Then, we analyzed the potential antiviral molecular mechanisms of FUM and GE through transcriptomics. However, there are still some limitations in our study. Firstly, the antiviral effects of FUM and GE are difficult to define precisely due to the high cost of the compounds. Secondly, to better evaluate the antiviral effect, it is necessary to do research in animal models. Finally, further mechanisms research of FUM and GE against RSV should be investigated both *in vitro* and *in vivo* models.

## Conclusion

In summary, we found that FUM and GE are novel inhibitors against RSV in multiple-cell models. Moreover, both compounds can reduce the inflammation level induced by RSV infection and protect mitochondrial function. Notably, FUM and GE have potentially broad-spectrum antiviral activity. Indicated that FUM and GE could be potential drug candidates for anti-RSV infection drug research.

## Data Availability

The datasets presented in this study can be found in online repositories. The names of the repository/repositories and accession number(s) can be found below: https://www.ncbi.nlm.nih.gov/sra/PRJNA1197729.
