# Supplementary material for: Fumarprotocetraric acid and geraniin were identified as novel inhibitors of human respiratory syncytial virus infection in vitro
Source: Front Cell Infect Microbiol. 2024 Dec 24;14:1484245. doi: 10.3389/fcimb.2024.1484245 (PMC11703719; doi:10.3389/fcimb.2024.1484245)
Supplement: Supplementary file 1 [file Table1.docx]

**Table. S1** The Real time-PCR primer sequences of cytokine detection.

| **Cytokine** | **Primer sequences** | |
| --- | --- | --- |
| IL1β- Forward | ATGATGGCTTATTACAGTGGCAA |  |
| IL1β- Reverse | GTCGGAGATTCGTAGCTGGA |  |
| IL-4- Forward | CGGCAACTTTGTCCACGGA |  |
| IL-4- Reverse | TCTGTTACGGTCAACTCGGTG |  |
| IL-6- Forward | ACTCACCTCTTCAGAACGAATTG |  |
| IL-6- Reverse | CCATCTTTGGAAGGTTCAGGTTG |  |
| IL-8 - Forward | TTTTGCCAAGGAGTGCTAAAGA |  |
| IL-8 - Reverse | AACCCTCTGCACCCAGTTTTC |  |
| TNF-α- Forward | GAGGCCAAGCCCTGGTATG |  |
| TNF-α- Reverse | CGGGCCGATTGATCTCAGC |  |
| MCP-1- Forward | CAGCCAGATGCAATCAATGCC |  |
| MCP-1- Reverse | TGGAATCCTGAACCCACTTCT |  |
| GAPDH- Forward | ACAACTTTGGTATCGTGGAAGG |  |
| GAPDH- Reverse | GCCATCACGCCACAGTTTC |  |
