# Supplementary material for: Fumarprotocetraric acid and geraniin were identified as novel inhibitors of human respiratory syncytial virus infection in vitro
Source: Front Cell Infect Microbiol. 2024 Dec 24;14:1484245. doi: 10.3389/fcimb.2024.1484245 (PMC11703719; doi:10.3389/fcimb.2024.1484245)
Supplement: Supplementary file 2 [file Table2.docx]

**Table. S2** The Real time-PCR primer sequences of DEGs.

| **Gene name** | **Primer sequences** | |
| --- | --- | --- |
| PI3- Forward | CACGGGAGTTCCTGTTAAAGG |  |
| PI3- Reverse | TCTTTCAAGCAGCGGTTAGGG |  |
| LTF- Forward | AGTCTACGGGACCGAAAGACA |  |
| LTF- Reverse | CAGACCTTGCAGTTCGTTCAG |  |
| DUOXA2- Forward | GTTCATAGGCGCAGAAATTGTG |  |
| DUOXA2- Reverse | AGACGGACACGGGCTGTAA |  |
| CSF3- Forward | GCTGCTTGAGCCAACTCCATA |  |
| CSF3- Reverse | GAACGCGGTACGACACCTC |  |
| SLC26A4- Forward | GCTCCCCAAATACCGAGTCAA |  |
| SLC26A4- Reverse | CACCACTGGAAAAGGTCCAAC |  |
| FCGBP- Forward | GCCAAGGCTGAGATGATAGGC |  |
| FCGBP- Reverse | CCTGCACAGAGATGGCATAGT |  |
| IFITM1- Forward | CCAAGGTCCACCGTGATTAAC |  |
| IFITM1- Reverse | ACCAGTTCAAGAAGAGGGTGTT |  |
| DOC2B- Forward | CAAATTCCGGCACAATGAGTTC |  |
| DOC2B- Reverse | GGACTTGTCTTCGGTCTTGTC |  |
| CXCL6- Forward | AGAGCTGCGTTGCACTTGTT |  |
| CXCL6- Reverse | GCAGTTTACCAATCGTTTTGGGG |  |
| C1S- Forward | ACTGTGCGTATGACTCAGTGC |  |
| C1S- Reverse | GGGGATTGTTACTGCTCCTCT |  |
| IFI6- Forward | GGTCTGCGATCCTGAATGGG |  |
| IFI6- Reverse | TCACTATCGAGATACTTGTGGGT |  |
| OASL- Forward | CCATTGTGCCTGCCTACAGAG |  |
| OASL- Reverse | CTTCAGCTTAGTTGGCCGATG |  |
| IFIT3- Forward | AAAAGCCCAACAACCCAGAAT |  |
| IFIT3- Reverse | CGTATTGGTTATCAGGACTCAGC |  |
| GAPDH- Forward | ACAACTTTGGTATCGTGGAAGG |  |
| GAPDH- Reverse | GCCATCACGCCACAGTTTC |  |
